# Concordance between PCR-based extraction-free saliva and nasopharyngeal swabs for SARS-CoV-2 testing

**DOI:** 10.12688/hrbopenres.13353.1

**Published:** 2021-07-30

**Authors:** Chiara De Santi, Benson Jacob, Patricia Kroich, Sean Doyle, Rebecca Ward, Brian Li, Owain Donnelly, Amy Dykes, Trisha Neelakant, David Neary, Ross McGuiness, Jacqueline Cafferkey, Kieran Ryan, Veronica Quadu, Killian McGrogan, Alejandro Garcia Leon, Patrick Mallon, Fidelma Fitzpatrick, Hilary Humphreys, Eoghan De Barra, Steve Kerrigan, Gianpiero L. Cavalleri

**Affiliations:** 1 School of Pharmacy and Biomolecular Sciences, Royal College of Surgeons in Ireland, Dublin, Ireland; 2 Department of International Health and Tropical Medicine, Royal College of Surgeons in Ireland, Dublin, Ireland; 3 Department of Microbiology, Beaumont Hospital, Dublin, Ireland; 4 Department of Surgical Affairs, Royal College of Surgeons in Ireland, Dublin, Ireland; 5 Mercer's Medical Centre, Royal College of Surgeons in Ireland, Dublin, Ireland; 6 Centre for Experimental Pathogen Host Research (CEPHR), University College Dublin, Dublin, Ireland; 7 Department of Clinical Microbiology, Royal College of Surgeons in Ireland, Dublin, Ireland; 8 Department of Infectious Diseases, Beaumont Hospital, Dublin, Ireland; 9 SFI FutureNeuro Research Centre, Royal College of Surgeons in Ireland, Dublin, Ireland

**Keywords:** SARS-CoV-2, Saliva, SalivaDirect, Nasopharyngeal swabs, RT-qPCR

## Abstract

**Introduction**: Saliva represents a less invasive alternative to nasopharyngeal swab (NPS) for severe acute respiratory syndrome coronavirus 2 (SARS-CoV-2) detection. SalivaDirect is a nucleic acid extraction-free method for detecting SARS-CoV2 in saliva specimens. Studies evaluating the concordance of gold standard NPS and newly developed SalivaDirect protocols are limited. The aim of our study was to to assess SalivaDirect as an alternative method for COVID-19 testing.

**Methods**: Matching NPS and saliva samples were analysed from a cohort of symptomatic (n=127) and asymptomatic (n=181) participants recruited from hospital and university settings, respectively. RNA was extracted from NPS while saliva samples were subjected to the SalivaDirect protocol before RT-qPCR analysis. The presence of SARS-Cov-2 was assessed using *RdRP* and *N1* gene targets in NPS and saliva, respectively.

**Results**: Overall we observed 94.3% sensitivity (95% CI 87.2-97.5%), and 95.9% specificity (95% CI 92.4-97.8%) in saliva when compared to matching NPS samples. Analysis of concordance demonstrated 95.5% accuracy overall for the saliva test relative to NPS, and a very high level of agreement (κ coefficient = 0.889, 95% CI 0.833–0.946) between the two sets of specimens. Fourteen of 308 samples were discordant, all from symptomatic patients. Ct values were >30 in 13/14 and >35 in 6/14 samples. No significant difference was found in the Ct values of matching NPS and saliva sample ( *p*=0.860). A highly significant correlation (r = 0.475, *p*<0.0001) was also found between the Ct values of the concordant positive saliva and NPS specimens.

**Conclusions**: Use of saliva processed according to the SalivaDirect protocol represents a valid method to detect SARS-CoV-2. Accurate and less invasive saliva screening is an attractive alternative to current testing methods based on NPS and would afford greater capacity to test asymptomatic populations especially in the context of frequent testing.

## Introduction

Severe acute respiratory syndrome coronavirus 2 (SARS-CoV-2) is a novel coronavirus that rapidly spread across the globe in late December 2019 and was declared a pandemic by the World Health Organization (WHO) in March 2020 [ press conference WHO 11 ^th^ March 2020]. Containing the spread of SARS-CoV-2 has been a significant challenge worldwide mainly because both asymptomatic and symptomatic individuals can transmit the virus ( Huff & Singh, 2020). A key approach to limiting cross-infection is robust testing and contact tracing. Currently, the gold standard for diagnosis is nucleic acid detection by reverse transcription quantitative PCR (RT-qPCR) ( Adhikari *et al.*, 2020). Nasopharyngeal swabs (NPS) were initially adopted as the preferred sampling procedure for SARS-CoV-2, due to established diagnostic practices for other respiratory infections ( Li *et al.*, 2013; Lieberman *et al.*, 2009). However, NPS are invasive and may induce coughing and sneezing, increasing the risk of transmission to healthcare professionals (HCP) conducting the procedure ( Kim *et al.*, 2017). NPS discomfort can also be a barrier to repeated, frequent testing as swabbing can induce effects that can last up to 24 hours post procedure including epistaxis, headaches, earaches and rhinorrhea ( Gupta *et al.*, 2021).

Testing for SARS-CoV-2 in saliva mitigates many of the challenges associated with NPS sampling ( Tan *et al.*, 2021; Vaz *et al.*, 2020; Wyllie *et al.*, 2020). Although various different protocols for SARS-CoV-2 testing in saliva have been proposed, including colorimetric reverse transcription loop-mediated isothermal amplification (RT-LAMP) and lateral flow assays ( Faustini *et al.*, 2020; Lalli *et al.*, 2021), RT-qPCR is the most common used modality ( Caulley *et al.*, 2021; Migueres *et al.*, 2020; Teo *et al.*, 2021) with a reported sensitivity between ~ 69 to 100% ( Azzi *et al.*, 2020; Kojima *et al.*, 2020; Pasomsub *et al.*, 2021; Skolimowska *et al.*, 2020; To *et al.*, 2020).

SalivaDirect is a nucleic acid extraction-free, cost effective and reliable method for detecting SARS-CoV2 which has been authorised for use by the FDA ( Vogels *et al.*, 2021, FDA press release 15 ^th^ August 2020). Using SalivaDirect, specimens can be self-collected in a sterile sample tube without a viral transport medium. Proteinase K addition and a short heat-treatment step precede RT-qPCR analysis ( Vogels *et al.*, 2021). Initial studies using SalivaDirect reported a significant positive agreement (94%) between paired saliva and NPS samples obtained from a hospital cohort of 37 asymptomatic HCP and 30 COVID-19-positive inpatients ( Vogels *et al.*, 2021). In another study, matched saliva and NPS samples obtained from 30 individuals with COVID-19 illustrated a 88.2% concordance when using the SalivaDirect protocol ( Rodríguez Flores *et al.*, 2021).

Data comparing the concordance of ‘gold standard’ NPS and the SalivaDirect protocol for ongoing testing as part of an infection prevention and control programme are limited. In the present study, we set out to compare matching NPS and extraction-free saliva samples for detection of SARS-CoV-2 viral RNA via RT-qPCR, to assess the suitability of saliva as an alternative specimen for COVID-19 testing. We studied two key demographics – asymptomatic university students and hospital inpatients admitted with respiratory symptoms due to COVID-19 related illnesses.

## Methods

### Ethical considerations

This study was approved by the Ethics Committee of Royal College of Surgeons in Ireland (RCSI) (study code: REC202010011) and the National Research Ethics Committee for COVID-19 (20-NREC-COV-056). All subjects involved in this study provided written informed consent.

### Sample collection

Research participants were recruited at two sites; a symptomatic patient cohort (n=127) was assembled at Beaumont Hospital, Dublin, Ireland and an asymptomatic ‘student’ cohort (n=181) at RCSI University of Medicine and Health Sciences, Dublin, Ireland. Recruitment took place during the period from November 2020 to March 2021 for the symptomatic population and in December 2020 and January 2021 for the asymptomatic student cohort.

Symptomatic individuals were inpatients at Beaumont Hospital who tested positive for SARS-CoV-2 on NPS-based admission testing, carried out in the hospital’s clinical microbiology laboratory (using either the CerTest Biotec VIASURE SARS-CoV-2 real time PCR detection kit or Cepheid Xpert Xpress SARS-CoV-2). Repeat NPS samples were taken during their hospital stay by a trained professional, followed by a same-day saliva sample collected by passive drooling. Asymptomatic students were recruited via an in-house screening programme at RCSI. NPS samples were collected by a trained professional with a saliva sample collected by passive drooling immediately afterwards. In both cohorts, exclusion criteria for participation to the study were any of the following activities conducted in the half hour prior to saliva sample collection: smoking, drinking any liquids, food, nasal sprays, tooth brushing and/or mouth washing.

### NPS sample processing

NPS from the symptomatic cohort were placed in 2 ml of Viral Transport Media and sent to the CEPHR Laboratory using a biomedical courier. Samples were aliquoted into 2 ml cryovials and stored at -80°C until further use. Using 250 µl of the biobanked NPS, RNA was extracted using the automated platform for nucleic acid extraction EX3600 (Liferiver Biotech, Shanghai, China) according to manufacturer’s instructions, with a nucleic acid elution volume of 60 µl. Following RNA extraction, samples underwent RT-qPCR for SARS-CoV-2 with primers directed against the RNA-dependent RNA polymerase ( *RdRp*) region of the viral genome, using the COVID-19 Genesig Real-Time PCR assay ( Primerdesign Ltd, Hampshire, United Kingdom) on The LightCycler 480 PCR platform ( Roche Diagnostics, Basel, Switzerland) with the following thermocycling conditions: 10 min 55°C, 2 min 95°C, 45 cycles of 10 sec 95°C/60 sec 60°C. Each run included a SARS-CoV-2 positive control (RNA), internal control, no-template control and a positive control of extraction (RNA from SARS-CoV-2 virus - 2019-nCoV/Italy-INMI1). Samples with quantification cycle (Cq) values below 40 cycles were defined as SARS-CoV-2-detected.

NPS from the asymptomatic cohort were processed in the COVID-19 Testing Lab at RCSI. RNA extraction for NPS samples was performed using a MagMax Viral/Pathogen II Nucleic Acid Isolation Kit ( ThermoFisher Scientific), as per manufacturers’ instructions on a KingFisher Flex Purification system (model 5400630, ThermoFisher Scientific) instrument using 200 µL of NPS sample input. RT-qPCR followed the Centers for Disease Control and Prevention (CDC) protocol ( Lu *et al.*, 2020). Briefly, amplification of the SARS-CoV-2 nucleocapsid gene ( *N1* and *N2)* and internal control ( *RP)* was performed using TaqPath 1-Step RT-qPCR Master Mix (4x, ThermoFisher Scientific) with the following sets of primers and probes: N1_F primer 5′- GACCCCAAAATCAGCGAAAT -3′ (500nM), N1_R primer 5′- TCTGGTTACTGCCAGTTGAATCTG -3′ (500nM), and N1_probe 5′-FAM-ACCCCGCATTACGTTTGGTGGACC -BHQ1-3′ (125nM); N2_F primer 5′- TTACAAACATTGGCCGCAAA -3′ (500nM), N2_R primer 5′- GCGCGACATTCCGAAGAA -3′ (500nM), and N2_probe 5′-Cy3- ACAATTTGCCCCCAGCGCTTCAG -BHQ1-3′ (125nM); RP_F primer 5′- AGATTTGGACCTGCGAGCG -3′ (500nM), RP_R primer 5′- GAGCGGCTGTCTCCACAAGT -3′ (500nM), and RP_probe 5′-Cy5- TTCTGACCTGAAGGCTCTGCGCG -BHQ1-3′ (125nM). The TaqPath RT-qPCR Master Mix (15 µL) was added to 5 µL of the RNA extracted from each NPS sample and run on a QuantStudio 7 (ThermoFisher Scientific) Pro Real-Time PCR with the following thermocycling conditions: 2 min 25°C, 15 min 50°C, 2 min 95°C and 45 cycles of 3 sec 95°C/30 sec 55°C. Cycle threshold (Ct) values lower than 40 was interpreted as detection of the gene and viral status of the clinical samples was called as per CDC recommendation ( Lu *et al.*, 2020).

### Saliva sample processing

All saliva specimens ( *i.e.* from both the symptomatic and asymptomatic cohorts) were processed in the COVID-19 Testing Laboratory at RCSI using the SalivaDirect protocol ( Vogels *et al.*, 2021). Briefly, 50 µl of each saliva sample was added to 2.5 µl of proteinase K, vortexed and incubated at 95ºC for 5 minutes to ensure inactivation of the virus. RT-qPCR amplification was performed using TaqPath 1-Step RT-qPCR Master Mix (4x, ThermoFisher Scientific), and the CDC *N1* and *RP* sets of primers and probes as per SalivaDirect protocol at the following concentrations: N1_F primer (400nM), N1_R primer (400nM), and N1_probe (200nM); RP_F primer (150nM), RP_R primer (150nM), and RP_probe (200nM). The TaqPath RT-qPCR Master Mix (15 µL) was added to 5 µL of the “extraction-free” saliva sample and run on a QuantStudio 7 Pro Real-Time PCR with the following thermocycling conditions: 10 min 52°C, 2 min 95°C and 45 cycles of 10 sec 95°C/30 sec 55°C. Ct values lower than 40 were interpreted as detection of the gene and viral status was called as per SalivaDirect recommendation ( Vogels *et al.*, 2021).

### RNA extraction from saliva and SarS-CoV-2 detection using TaqPath COVID-19 CE-IVD RT-PCR Kit

In some cases, i.e. when the saliva and NPS testing results were not concordant, saliva samples were extracted using the MagMax Viral/Pathogen II Nucleic Acid Isolation Kit (ThermoFisher Scientific) following the protocol described above for NPS processing. This was a quality control step introduced to reduce the risk of false positive or negative results from saliva processed according to SalivaDirect protocol. RT-qPCR amplification of the SARS-CoV-2 *ORF1* gene, *N* gene and *S* gene was performed using TaqPath 1‑Step Multiplex Master Mix (No ROX) (4X) and the TaqPath COVID-19 CE-IVD RT-PCR Kit as per manufacturers’ instructions. Briefly, 15 µL of the prepared mix were added to 10 µL of the RNA extracted from each saliva sample and run on a QuantStudio 7 Pro Real-Time PCR with the following thermocycling conditions: 2 min 25°C, 10 min 53°C, 2 min 95°C, and 40 cycles of 3 sec 95°C/30 sec 60°C. Ct values lower than 37 were interpreted as indicating the expression of that gene and viral status was called as per TaqPath COVID-19 CE-IVD RT-PCR Kit manufacturers’ instructions.

### Statistical analyses

All statistical analyses were performed using GraphPad PRISM version 8.0.0 (GraphPad Software, San Diego, CA, USA). Sensitivity, specificity and 95% confidence intervals (CI) were calculated to assess diagnostic performance. Agreement between the NPS and saliva specimens for the virus detection ability was also assessed using Cohen’s Kappa (κ coefficient). Paired *t*-tests were used to compare the Ct values between NPS and saliva. Correlation between NPS and saliva Ct values were quantified using the Pearson correlation coefficient (Pearson *r*). All *p*-values were two-sided and *p* <0.05 was considered significant.

## Results

A total of 308 individuals, 181 asymptomatic students and 127 symptomatic patients, were included in this study ( Table 1). 

**Table 1.** Demographic and clinical characteristics of the symptomatic (n=127) and asymptomatic (n=181) cohorts. For the symptomatic patients, we report the maximum COVID-19 disease severity (as per WHO guidelines) reached during their hospital stay.

		Hospitalised patients (n=127)	Students (n=181)
Sex (%)	Male	44.9	45.4
	Female	37.8	52.4
	Unknown	17.3	2.2
Age (years)	Median (IQR ^$^)	68 (26)	24 (3)
Clinical Phenotype (%)		Mild: 31.5	Asymptomatic: 100
		Moderate: 20.5	
		Severe: 18.9	
		Critical *: 9.4	
		Unknown: 19.7	

^$^Interquantile Range; *Critical COVID-19 severity score included both sepsis (n=3) and acute respiratory distress syndrome (ARDS) (n=9).

We first tested the correlation between NPS and saliva testing results in the symptomatic cohort. SARS-CoV-2 was detected in 86 of 127 patient NPS, while it was undetected in 41. Saliva testing in the same cohort indicated that 90 patients tested positive for SARS-CoV-2, while 37 tested negative. The sensitivity of saliva compared to NPS was 94.2% (95% CI 87.1-97.4%), while the specificity was 78.1% (95% CI 63.3-88%) in our symptomatic cohort.

We next tested the correlation between NPS and saliva in the asymptomatic cohort. Of 181 students, one tested positive both in NPS and saliva samples, while 180 tested negative in both. Sensitivity (95% CI 5-100%) and specificity (95% CI 97.9-100%) were therefore both 100% in this cohort.

The overall concordance between NPS and saliva testing is shown in Table 2. In the combined cohort, the sensitivity of saliva compared to NPS was 94.3% (95% CI 87.2-97.5%), while the specificity was 95.9% (95% CI 92.4-97.8%). Analysis of the concordance between the NPS and saliva specimens demonstrated an overall 95.5% accuracy for the saliva test relative to NPS and a very high level of agreement (κ coefficient = 0.889, 95% CI 0.833–0.946) between the two specimens. Nonetheless, we found 14 discordant samples between saliva and NPS. SARS-CoV-2 was detected in nine saliva samples but not detected in their matched NPS. On the other hand, viral RNA was not detected in five saliva samples but detected in the matched NPS. All 14 discordant samples belonged to the symptomatic hospitalised cohort. Ct values were >30 in 13/14 of these discordant samples and >35 in 6/14 samples.

**Table 2.** Diagnostic performance of SalivaDirect protocol compared to the gold standard NPS testing in a symptomatic (n=127) and asymptomatic (n=181) cohorts.

		Nasopharyngeal swab	
		Positive	Negative
Salivadirect – Saliva	Positive	82	9
	Negative	5	212
Total		87	221
Positive agreement = 94.3 % (95 % CI 87.2-97.5 %)			
Negative agreement = 95.9 % (95 % CI 92.4-97.8 %)			

To further explore the discordant results, we extracted RNA from the 14 discordant saliva samples and retested the samples with the TaqPath COVID-19 CE-IVD RT-PCR kit. SARS-CoV-2 viral RNA detection was confirmed in 7/9 ‘false positive’ saliva samples, indicating these were actually true positives which the NPS test failed to detect. The remaining two saliva samples in which SARS-CoV-2 was undetected had Ct values ≥35 using the SalivaDirect protocol. All five ‘false negative’ also tested negative using the TaqPath protocol, indicating that in this case saliva testing failed to detect the virus which was instead detected by NPS testing. The *RdRp* Ct values from NPS from these samples were >30 using the Genesig Real-Time PCR assay.

**Figure 1.**
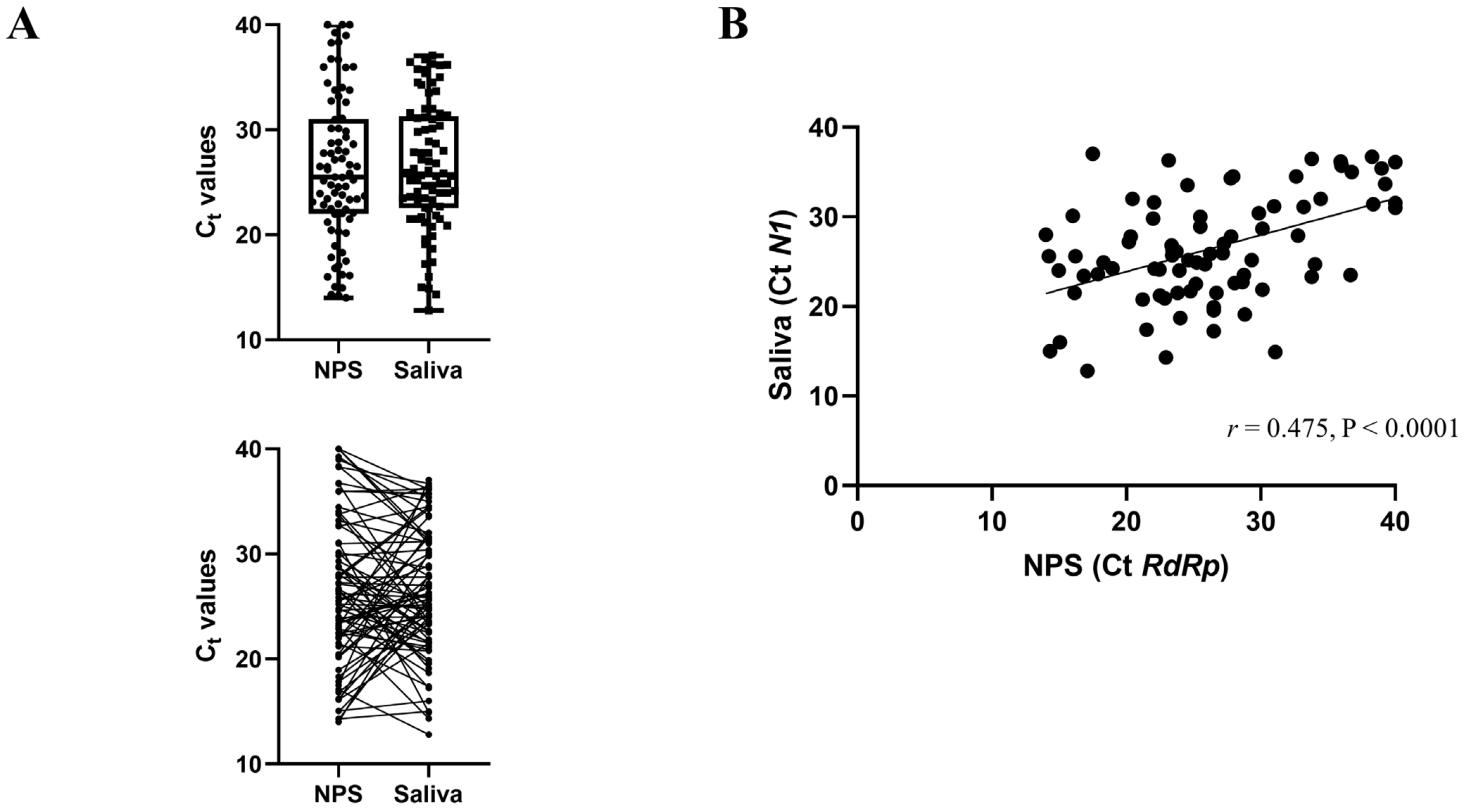
Comparison of Ct values from concordant positive saliva and NPS samples from the symptomatic cohort (n=81). ( **A**) The mean Ct values for saliva specimens are not significantly different than the mean for NPS specimens. The lines indicate samples from the same patient. ( **B**) Correlation of Ct values between saliva ( *N1*) and NPS ( *RdRp*) PCR. The scatter plot shows the comparison of Ct values between the two methods.

We next compared the Ct values of the concordant positive saliva and NPS samples ( Figure 1A) in the symptomatic cohort. *N1* Ct values were reported for saliva samples according to SalivaDirect protocol, and *RdRp* Ct values for the NPS were reported, as it is the target gene of the Genesig Real-Time PCR assay. The overall mean and standard deviation (SD) Ct value for the positive NPS specimens and saliva samples was 26.36 (SD 7.03) and 26.49 (SD 6.04), respectively. The difference in mean Ct values (0.132) was not statistically significant ( *p*=0.860). We also found a highly significant correlation between the Ct values of the positive saliva and NPS specimens (Pearson *r* = 0.475, *p*<0.0001, Figure 1B).

**Figure 2.**
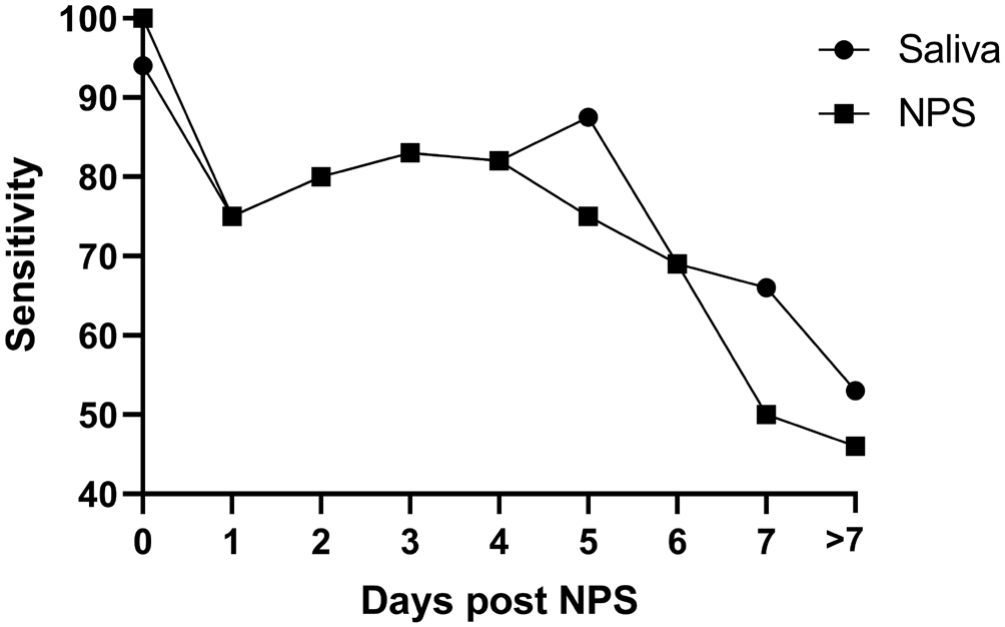
Sensitivity (in %) of saliva and repeat NPS in the symptomatic cohort (n=127). X axis: the delay (in days) between the positive NPS on hospital admission and the collection of the matching saliva and NPS sample. Matching NPS/saliva samples taken > 7 days post admission were pooled and reported as ‘>7’ (average of 11.53 days, 95% CI 10.02-13.03).

Finally, we compared NPS and saliva test results obtained from the hospitalised symptomatic patients (127 individuals) with the results from their initial positive SARS-CoV-2 swab, which was performed on admission to hospital. All these individuals provided one matching research NPS/saliva sample during their admission period, an average of 4.73 days (95% CI 4.05-5.42) post initial diagnosis. As expected, the positive agreement of NPS and saliva testing decreased with time, but a similar pattern for saliva and NPS is notable (see Figure 2).

## Discussion

Use of saliva to detect SARS-CoV-2 represents a valid and accurate alternative to NPS sampling. Our results indicate a 94.3% sensitivity and 95.9% specificity of saliva when compared to a matching NPS taken on the same day in a combined cohort of symptomatic and asymptomatic individuals. Our results are consistent with the original publication of the SalivaDirect method, where the positive agreement was 94% in the hospitalised cohort, and sensitivity and specificity of saliva versus NPS in 3779 asymptomatic individuals were 89.5% and >99.9%, respectively ( Vogels *et al.*, 2021). A separate study reported a sensitivity of 88.2% of saliva samples assessed with SalivaDirect when compared to matching NPS samples taken from 30 COVID-19-positive individuals ( Rodríguez Flores *et al.*, 2021).

In samples where the virus was detected, we observed a strong correlation between viral gene Ct values across NPS and saliva samples, consistent with recent reports ( Braz-Silva *et al.*, 2020; Mahmoud *et al.*, 2021).

Although we found an overall accuracy of 95.5%, 14 samples from the symptomatic cohort were discordant between matching NPS and saliva samples, with five individuals testing positive by NPS only and nine by saliva only. The majority of these samples showed Ct values (of either *RdRp* for NPS or *N1* for saliva) over 30, suggesting that a higher discordance between NPS and saliva testing results could be observed for high Ct values, as reported before ( Kandel *et al.*, 2020). When we extracted RNA from the 14 discordant saliva samples and tested them using an alternative PCR protocol, we found two ‘false positives’ in saliva. This confirmatory step in case of high Ct values from the SalivaDirect protocol could therefore be helpful to avoid false positives, which are proportionally greater in low prevalence settings ( Basile *et al.*, 2020; Healy *et al.*, 2021).

In the hospitalised symptomatic cohort, we assessed the positivity rate in NPS, and saliva samples taken a variable amount of time after the first diagnosis. As expected, the positive agreement between the first diagnosis and the saliva or repeat NPS decreased over time, reflecting recovery and viral clearance. These findings are consistent with other longitudinal studies of COVID-19 testing ( Smith *et al.*, 2021; Wyllie *et al.*, 2020). However, our results are the first to show that the drop in performance is consistent across saliva and NPS, thereby showing once again that the SalivaDirect accuracy is very similar to the gold-standard RT-qPCR from NPS.

Limitations of the current study include the presence of only one positive in the asymptomatic population which limits the value of the sensitivity calculation in that cohort. In addition, a more closely matched control group would have been beneficial in this study, as samples from a healthy student population were compared to the symptomatic hospitalised cohort. Lastly, the use of Ct values shows a trend of the viral load but does not allow exact quantification of viral copies/ml due to the absence of a standard curve included in the RT-qPCR analyses.

Overall, although the sensitivity and specificity are slightly lower, this work suggests that the SalivaDirect protocol represents a valid alternative to NPS. Other diagnostic assays are available including lateral flow antigen tests (LFAT); however, a pilot study applying LFAT in an asymptomatic population in the UK indicated a sensitivity of only ~49% ( Wise, 2020). Saliva is a less invasive and hence a more acceptable sampling method compared to NPS. ( Goldfarb *et al.*, 2021; Kinloch *et al.*, 2020). In addition, saliva collection does not require direct interaction between HCP and individuals. This offers several advantages, decreasing the potential for cross-infection as well as alleviating testing bottlenecks by decreasing the need for qualified HCP, personal protective equipment, and swab supply ( Wyllie *et al.*, 2020). Saliva can be self-collected, although individuals should be instructed in proper use of the self-collection tube, including its decontamination after saliva collection. These advantages are particularly relevant in the context of a surveillance programme where compliance rates play a crucial role in the long-term success of the screening initiative and individuals would benefit from a less invasive and accurate sampling method, easier to collect in a serial manner.

## Data availability

DRYAD: Concordance between PCR-based extraction-free saliva and nasopharyngeal swabs for SARS-CoV-2 testing, https://doi.org/10.5061/dryad.ksn02v74n ( De Santi, 2021)

This project contains the following underlying data:

1. Ct_values_for_matched_NPS_and_saliva_samples_(asymptomatic_cohort).xlsx.This table shows the Ct values of *N1* (both for NPS and saliva) in the samples belonging to the asymptomatic cohort (n=181 students).
2. Ct_values_for_matched_NPS_and_saliva_samples_(symptomatic_cohort).xlsx. This table shows the Ct values of *RdRp* (NPS) and *N1* (saliva) in the samples belonging to the symptomatic cohort (n=127 hospitalised symptomatic individuals).

Data are available under the terms of the Creative Commons Zero "No rights reserved" data waiver (CC0 1.0 Public domain dedication).
