# Supplementary material for: Concordance between PCR-based extraction-free saliva and nasopharyngeal swabs for SARS-CoV-2 testing
Source: HRB Open Res. 2021 Oct 15;4:85. Originally published 2021 Jul 30. [Version 2] doi: 10.12688/hrbopenres.13353.2 (PMC8408542; doi:10.12688/hrbopenres.13353.2)
Supplement: Peer review history

# Peer review: Reviewer response for version 1

**DOI:** 10.21956/hrbopenres.14541.r29961

**Published:** 2021-08-31

**Authors:** Mario A. Rodríguez-Pérez, Luis Mario Rodríguez-Martínez, Sofia Nohemí Rodríguez-Flores

**Affiliations:** 1 Instituto Politécnico Nacional. Centro de Biotecnología Genómica. Reynosa, México., Centro de Biotecnología Genómica, Reynosa, Mexico; 2 Instituto Politécnico Nacional, Centro de Biotecnología Genómica, Reynosa, Mexico; 3 Instituto Politécnico Nacional, Unidad de Desarrollo e Investigación en Bioprocesos, Escuela de Ciencias Biológicas, Ciudad de México, Mexico

**General comments**

 The present study compares NPS and Saliva as a COVID-19 sample source. The study included the diagnosis of a “cohort” of 127 symptomatic participants and 181 asymptomatic participants with COVID-19; however, it is not clear how many samples were taken per patient, the date (day), and the viral loads expressed as CTs. Figure 2 depicts that the sampling was conducted several times; however, in fragments of the writing text it appears that only one sample was taken per participant; thus, it would not be a cohort as it is from a longitudinal study.  Instead, this study would be cross-sectional since the sample was taken per participant in a single moment.  The authors should clarify which is correct.

 The number of samples evaluated of 308 is enough to widely support the findings: 94.3% sensitivity and specificity of 95.9%.

 In this comparison study, the authors have evaluated two systems:  Firstly, the CDC protocols using RT-qPCR with N1, N2 genes, and RP for SARS-CoV-2; Secondly, if the sample was not concordant in its result, it was evaluated with the TaqPath COVID-19 CE-IVD RT-PCR kit, which uses the ORF, N, and S genes as a confirmatory test. Finally, this implied a robust and reliable result; this was done for the 14 non concordant (discordant) samples found.

**Particular comments and edits**

**Methods**

 Saliva sample processing

 “Briefly, 50 µl of each saliva sample was added to 2.5 µl of proteinase K”- Mention the concentration or units of proteinase K added and Brand.

 Mention about RT-qPCR cycle changes with NPS and saliva.

 “RNA extraction from saliva and SarS-CoV-2 detection using TaqPath COVID-19 CE-IVD RT-PCR Kit”. Correct in subtitle "SarS-CoV-2" to "SARS-CoV-2"

**Results**

 “Sensitivity (95% CI 5-100%) and specificity (95% CI 97.9-100%) were, therefore, both 100% in this cohort.”

 95%-CIs surrounding the point estimator of sensitivity is too wide (5-100%). Re-calculate values or clarify.

 Table 2 could be improved, for example, place 3 columns indicating detection method, positive, and negative. Transfer results of NPS into a row.

 Figure 2 in the Y-axis named as "Sensitivity" correct to "Sensitivity (%).

 In the Figure´s legend, instead of "Sensitivity (in%) ..." correct to "Percentage of sensitivity ..."

**Discussion**

 Discuss what it may imply that by testing saliva this does not meet the established requirements (smoking, drinking fluids, etc.)

 It should be compared to currently used methods for rapid detection showing over 90% sensitivity.  Why use SalivaDirect and not those?

 Did the authors notice any disadvantages of using the SalivaDirect? If so, discuss it.

Is the work clearly and accurately presented and does it cite the current literature?

Yes

If applicable, is the statistical analysis and its interpretation appropriate?

Yes

Are all the source data underlying the results available to ensure full reproducibility?

Yes

Is the study design appropriate and is the work technically sound?

Yes

Are the conclusions drawn adequately supported by the results?

Yes

Are sufficient details of methods and analysis provided to allow replication by others?

Yes

Reviewer Expertise:

Epidemiology

We confirm that we have read this submission and believe that we have an appropriate level of expertise to confirm that it is of an acceptable scientific standard.

# Response

**Published:** 2021-10-01

**Authors:** Chiara De Santi

**Affiliations:** Royal College of Surgeons in Ireland, Ireland

We thank the reviewers for their summary, comments and suggestions. Please find a point-by-point answer to the reviewers' queries below:

**General comments**

*The present study compares NPS and Saliva as a COVID-19 sample source. The study included the diagnosis of a “cohort” of 127 symptomatic participants and 181 asymptomatic participants with COVID-19; however, it is not clear how many samples were taken per patient, the date (day), and the viral loads expressed as CTs. Figure 2 depicts that the sampling was conducted several times; however, in fragments of the writing text it appears that only one sample was taken per participant; thus, it would not be a cohort as it is from a longitudinal study. Instead, this study would be cross-sectional since the sample was taken per participant in a single moment. The authors should clarify which is correct.*

*The number of samples evaluated of 308 is enough to widely support the findings: 94.3% sensitivity and specificity of 95.9%.*

*In this comparison study, the authors have evaluated two systems: Firstly, the CDC protocols using RT-qPCR with N1, N2 genes, and RP for SARS-CoV-2; Secondly, if the sample was not concordant in its result, it was evaluated with the TaqPath COVID-19 CE-IVD RT-PCR kit, which uses the ORF, N, and S genes as a confirmatory test. Finally, this implied a robust and reliable result; this was done for the 14 non concordant (discordant) samples found.*

**Answer:** We thank the reviewer for their summary and comments. In response to the comment on the longitudinal vs cross-sectional study definition, we have now added explanatory sentences both in the methods and results section. Briefly, the symptomatic cohort consisted of inpatients who provided a first NPS sample on admission to hospital (tested in the hospital laboratories). They were recruited to the study while in hospital and provided a second NPS sample on the same day as the saliva sample, both available for research purposes/the current study. Therefore, we consider this section of the work a longitudinal study. However, the asymptomatic cohort (students) only provided one same day NPS/saliva sample during their appointment as per routine in-house screening at our University (RCSI).

**Methods**

*Saliva sample processing*

*“Briefly, 50 μl of each saliva sample was added to 2.5 μl of proteinase K”- Mention the concentration or units of proteinase K added and Brand.*

**Answer**: This detail has now been added to the methods section text.

*Mention about RT-qPCR cycle changes with NPS and saliva.*

**Answer:** The comparison between Cts from concordant NPS and saliva samples (for the symptomatic cohort) is addressed in the results section (“We next compared the Ct values of the concordant positive saliva and NPS samples (Figure 1A) in the symptomatic cohort…”) and shown in Figure 1. Although we did observe strong correlation between the Ct values in NSP and saliva as expected from the literature, we did not see significant differences in Ct count as other relevant publications have reported. This point has not been added to the discussion section, where we mention that it should be noted that we tested for different viral genes for NPS ( *RdRp*) and saliva ( *N1*).

*“RNA extraction from saliva and SarS-CoV-2 detection using TaqPath COVID-19 CE-IVD RT-PCR Kit”. Correct in subtitle "SarS-CoV-2" to "SARS-CoV-2"*

**Answer:** This has now been changed in the method section.

**Results**

*“Sensitivity (95% CI 5-100%) and specificity (95% CI 97.9-100%) were, therefore, both 100% in this*

*cohort.” 95%-CIs surrounding the point estimator of sensitivity is too wide (5-100%). Re-calculate values or*

*clarify.*

**Answer:** The 95%-CIs surrounding the sensitivity ( *CI 5-100%)* is correctly calculated. The wide interval is to be expected given that only one student tested positive in our asymptomatic cohort. We acknowledge in the discussion the limited value of this sensitivity value. We therefore combined the two cohorts of symptomatic and asymptomatic for the overall sensitivity (94.3%; 95% CI 87.2-97.5%) and specificity values (95.9%; 95% CI 92.4-97.8%).

*Table 2 could be improved, for example, place 3 columns indicating detection method, positive, and negative. Transfer results of NPS into a row.*

**Answer:** The data in Table 2 regarding sensitivities and sensitivities of SalivaDirect when compared to NPS are presented in the same format as presented in several publications (Skolimowska *et al*., 2020; Braz-Silva *et al*., 2020; Mahmoud *et al*., 2021; Pasomsub *et al*., 2021 etc) and we think that consistency allows for the easiest comparison of various tests for the readers.

*Figure 2 in the Y-axis named as "Sensitivity" correct to "Sensitivity (%). In the Figure´s legend, instead of "Sensitivity (in%) ..." correct to "Percentage of sensitivity ..."*

**Answer: ** The suggested changes have been made to figure 2 and associated legend.

**Discussion**

*Discuss what it may imply that by testing saliva this does not meet the established requirements (smoking, drinking fluids, etc.).*

**Answer:** Smoking, drinking any liquids, food, nasal sprays, tooth brushing and/or mouth washing might affect the quality of the saliva sample and/or the validity of the saliva testing. For example, the use of mouth wash was shown to reduce the viral load in saliva samples (Seneviratne *et al*., 2021) and therefore using mouth wash prior to providing a saliva sample should be avoided. Presence of food, mucus and residues such as those from brushing teeth or smoking can decrease the quality of the samples and prove their handling more difficult due to particles presence in the saliva. A sentence about the effects of these activities on saliva testing has now been added to the method section.

*It should be compared to currently used methods for rapid detection showing over 90% sensitivity. Why use SalivaDirect and not those?*

**Answer:** We thank the reviewer for this comment. In the general context, it has been well established that rapid antigen testing have a high sensitivity (over 90%) in case of high viral load/low Ct values (although antigen approaches were not tested in parallel in our cohorts). For example, Van der Moeren and co-authors showed that BD Veritor System for Rapid Detection of SARS-CoV-2 has an overall sensitivity of 78.9% and reaching 93% for samples with Cts lower than 30 (Van der Moeren N, *et al.* PLoS One. 2021. PMID: 33983971). In another study, Fernandez-Montero reported a sensitivity of Roche SARS-CoV-2 Rapid Antigen Test of 71.43%, which increases to 93.75% when Cts were lower than 25 (Fernandez-Montero *et al*., EClinicalMedicine. 2021 Jul;37:100954. doi: 10.1016/j.eclinm.2021.100954, PMID: 34127960). In our hands the sensitivity of saliva testing compared to NPS was maintained for high Cts – it was 100% for Cts in NPS lower than 30, and, as reported in our manuscript, 94.3% overall. But most importantly, the rapid antigen tests currently available still required a nasopharyngeal swab (often self-administered in case of asymptomatic screening programmes) which is widely perceived as uncomfortable and linked to coughing, sneezing, headache which can be a barrier to repeated testing (for example with younger students). Therefore, we propose PCR-based saliva testing as a valid alternative to NSP, especially when employing SalivaDirect protocol which skips the RNA extraction step (thereby saving money and time), is a relatively cheap and accurate method to detect SARS-CoV-2, especially for those individuals who will require repeated testing. Moreover, reproducible finding of PCR-based saliva testing is a prerequisite to developing and validating rapid antigen saliva tests, which so far haven’t reached satisfactory diagnostic performance (Nagura-Ikeda *et al.*, J Clin Microbiol. 2020 Aug 24;58(9):e01438-20; Audigé *et al*., Microorganisms. 2021 Aug 10;9(8):1700). We have now added a sentence in the discussion section that reinforce the advantages of this approach for PCR-based SARS-CoV-2 testing.

*Did the authors notice any disadvantages of using the SalivaDirect? If so, discuss it.*

**Answer:** We found no significant disadvantage in using SalivaDirect compared to RNA extraction-based saliva testing. However we did notice that the handling of the saliva samples was more challenging than the NPS (they were more gloopy, the colour and density varied greatly among the samples). We have now mention this in the methods section.

# Peer review: Reviewer response for version 1

**DOI:** 10.21956/hrbopenres.14541.r30007

**Published:** 2021-08-19

**Authors:** Stephanie Russo Perniciaro

**Affiliations:** 1 Department of Epidemiology of Microbial Diseases, Public Health Modeling Unit, Yale School of Public Health, Yale University, New Haven, CT, USA

This paper describes and contextualizes the results of a study comparing nasopharyngeal swab samples for SARS-CoV-2 detection with saliva samples for SARS-CoV-2 detection. The authors enrolled a symptomatic cohort and an asymptomatic cohort, and observed high concordance between nasopharyngeal swabs and saliva samples. Out of ~300 total samples, 14 were discordant. The sensitivity and specificity of saliva testing with the SalivaDirect protocol were high, similar to other studies of the implementation of this low-cost protocol.

 The authors' analysis is thorough and appropriate for the data. Having only one person with a positive test in one of the two study cohort populations makes full generalizability of the results difficult, but the authors address this and contextualize it appropriately.

Is the work clearly and accurately presented and does it cite the current literature?

Yes

If applicable, is the statistical analysis and its interpretation appropriate?

Yes

Are all the source data underlying the results available to ensure full reproducibility?

Yes

Is the study design appropriate and is the work technically sound?

Yes

Are the conclusions drawn adequately supported by the results?

Yes

Are sufficient details of methods and analysis provided to allow replication by others?

Yes

Reviewer Expertise:

Epidemiology, infectious diseases, respiratory infections

I confirm that I have read this submission and believe that I have an appropriate level of expertise to confirm that it is of an acceptable scientific standard.
